# Pathergy beyond neutrophilic dermatoses: A case of localized leukemia cutis at the site of intravenous infiltration

**DOI:** 10.1016/j.jdcr.2025.06.068

**Published:** 2025-10-08

**Authors:** Janet Choi, Donald Lei, Solbie Choi, Jasmine H. Wong, Hatice Zengin, Bijal Amin, Benedict Wu

**Affiliations:** a Division of Dermatology, Department of Medicine, Albert Einstein College of Medicine/Montefiore Medical Center, Bronx, New York; b Department of Pathology, Albert Einstein College of Medicine/Montefiore Medical Center, Bronx, New York

**Keywords:** acute myeloid leukemia, intravenous infiltration, leukemia cutis, pathergy

*To the Editor:* We greatly appreciated Agrawal et al’s report of leukemia cutis (LC) following trauma from plant thorns in a newly diagnosed leukemia patient.^1^ LC is a rare manifestation of systemic leukemia caused by neoplastic leukocytes infiltrating the skin. We present another unusual case of LC developing at a traumatized site in a newly diagnosed leukemia patient.

A 41-year-old man with pancytopenia and newly diagnosed acute myeloid leukemia, admitted for induction chemotherapy, presented with 2 enlarging, painful, and violaceous papulonodules at the site of a prior intravenous infiltration in the left antecubital fossa (Fig 1). The distal lesion had a brown, hyperkeratotic crust with underlying purulence. Given the patient’s immunocompromised state, an infectious process such as an opportunistic fungal or angioinvasive bacterial infection was initially suspected.^2^

**Fig 1:**
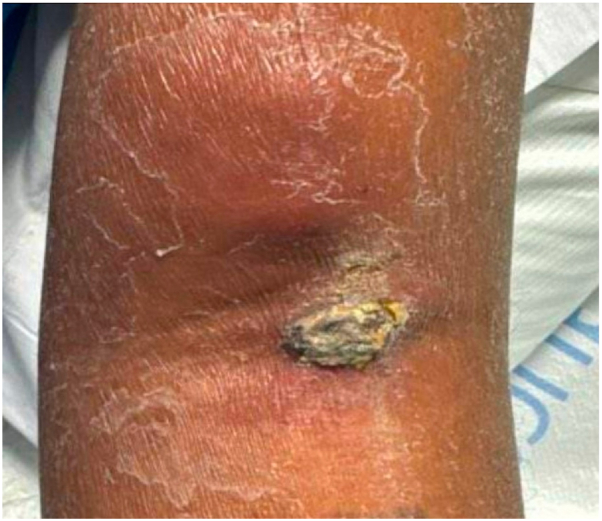
Clinical image of leukemia cutis on the left antecubital fossa.

Two punch biopsies for histology and tissue cultures were collected. The tissue culture grew methicillin-susceptible *Staphylococcus aureus,* and intravenous cefazolin was initiated. However, the skin biopsy revealed atypical cells in the dermis expressing CD33 (Fig 2, *A*-*C*), CD117, and myeloperoxidase, consistent with LC.

**Fig 2:**
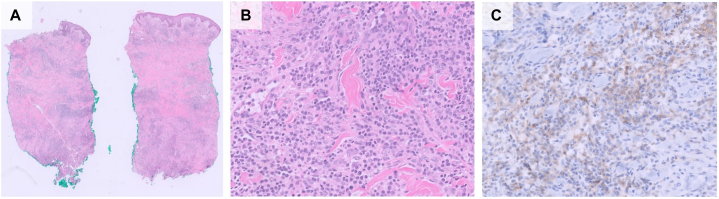
Histopathology showing leukemia cutis. There is dermal infiltration of blasts with round nuclei, open chromatin, visible nucleoli, and a moderate amount of cytoplasm, with scattered inflammatory cells such as small lymphocytes and histiocytes. The majority of the cells expressed CD33. **A,** H&E, 1×, **(B)** H&E, 20×, and **(C)** CD33.

Pathergy, a hypersensitivity reaction to minor trauma seen in neutrophilic dermatoses, is rarely reported with LC.^3^ To our knowledge, only 1 other case in the literature describes LC developing at a site of trauma in an acute myeloid leukemia patient.^4^ Our case further underscores the importance of histopathologic evaluation of unusual skin lesions arising after trauma in patients with underlying hematologic malignancies.

## Conflicts of interest

None disclosed.
